# The effectiveness of two occupational health intervention programmes in reducing sickness absence among employees at risk. Two randomised controlled trials

**DOI:** 10.1136/oem.2007.032706

**Published:** 2007-08-06

**Authors:** S Taimela, A Malmivaara, S Justén, E Läärä, H Sintonen, J Tiekso, T Aro

**Affiliations:** 1 Evalua International, Vantaa, Finland; 2 Finnish Office for Health Technology Assessment, FinOHTA/Stakes, Helsinki, Finland; 3 University of Oulu, Department of Mathematical Sciences, Oulu, Finland; 4 University of Helsinki, Department of Public Health, and FinOHTA, Helsinki, Finland; 5 Mutual Pension Insurance Company Ilmarinen, Helsinki, Finland

## Abstract

**Objectives:**

To evaluate the effectiveness of two occupational health intervention programmes, both compared with usual care.

**Methods:**

Based on a health survey, 1341 employees (88% males) in construction, service and maintenance work were classified into three groups: “low risk” (n = 386), “intermediate risk” (n = 537) and “high risk” (n = 418) of sickness absence. Two separate randomised trials were performed in the groups “high risk” and “intermediate risk”, respectively. Those high risk subjects that were allocated to the intervention group (n = 209) were invited to occupational health service for a consultation. The intervention included, if appropriate, a referral to specialist treatment. Among the intermediate risk employees those in the intervention group (n = 268) were invited to call a phone advice centre. In both trials the control group received usual occupational health care. The primary outcome was sickness absence during a 12-month follow-up (register data).

**Results:**

The high risk group, representing 31% of the cohort, accounted for 62% of sickness absence days. In the trial for the high risk group the mean sickness absence was 30 days in the usual care group and 19 days in the intervention group; the mean difference was 11 days (95% CI 1 to 20 days). In the trial for the intermediate risk group the mean sickness absence was 7 days in both arms (95% CI of the mean difference –2.3 to 2.4 days).

**Conclusions:**

The identification of high risk of work disability was successful. The occupational health intervention was effective in controlling work loss to a degree that is likely to be economically advantageous within the high risk group. The phone advice intervention for the intermediate risk group was not effective in controlling work loss primarily due to poor adherence.

Sickness absence may result in considerable personal and public financial consequences. Long-term sickness absence also predicts early retirement.^1^^–^3 Biographical and socioeconomic factors, diagnosed diseases, poor self-rated health, chronic complaints and poor work capacity predict sickness absence.^4^^–^7

Some randomised controlled trials (RCTs) have been performed in occupational settings in order to intervene specific diseases (musculoskeletal disorders or depression), or to advocate exercise.^8^^–^14 Only a few studies have aimed at identifying employees at high risk of work disability^7^ ^15^ or at reducing sickness absence within a high risk subgroup.^16^ ^17^

Telephone health counselling has been marketed as a low-cost intervention, but its efficacy in the occupational healthcare setting has not been tested in a randomised trial.

In this study we evaluated the effectiveness of two interventions for employees at high or intermediate risk of sickness absence, respectively. Subjects in the intervention for high risk were invited to a consultation at the occupational health services. Subjects in the intervention for intermediate risk were invited to call a telephone health advice centre.

## METHODS

### Study design and ethics

The design was a longitudinal cohort study with two embedded randomised trials. The risk of work disability was classified with self-administered questionnaires.^18^ Table 1 shows the criteria for the risk classification. Two separate randomised trials were performed in the subgroups of “high risk” (HR) and “intermediate risk” (IR) of sickness absence, respectively. The primary outcome was sickness absence during the 12-month follow-up. The Helsinki University Research Ethics Board approved the study, and it was performed according to the Declaration of Helsinki.

**Table 1:** The criteria for classifying the employees into “high risk” and “intermediate risk” groups

Topic	Criteria
“High risk” for work disability	At least one of the criteria fulfilled
Impairment due to musculoskeletal problems at work^19^	⩾5 (scale 0–10)
Potential depression^20^	DEPS score ⩾11 (scale 0–30)
Distress^21^	“Very much” feeling tense, strained, nervous and/or anxious because things are on one’s mind all the time
Fatigue^19^	“Very much” feeling of being squeezed empty because of work
Sleep disturbances^22^	Problems in falling asleep or night awakenings AND daytime tiredness daily or almost daily
Future working ability^23^	Uncertain of own ability or quite sure of not being able to continue in the present job due to health problems
“Intermediate risk” for work disability	At least one of the criteria fulfilled, but none of the criteria for “high risk” fulfilled
Impairment due to musculoskeletal problems at work^19^	4 (scale 0–10)
Impairment due to musculoskeletal problems at leisure time activities^19^	⩾5 (scale 0–10)
Pain (frequency and intensity)	At least “moderate” pain that “affects working ability” at minimum three times a week
Weight problems	BMI (body mass index) ⩾30 or BMI ⩽18.5
Excess alcohol consumption^24^	Males ⩾350 ml/week; Females ⩾240 ml/week*
Mood disturbances^20^	DEPS score ⩾8, (scale 0–30)
Sleep disturbances^22^	Problems in falling asleep or night awakenings AND daytime tiredness three times a week or more
Daytime sleepiness^25^	Epworth sleepiness scale (ESS) score ⩾8 (scale 0–24)
Suspicion of sleep apnoea^22^	Snoring and shortness of breath while asleep daily or almost daily
Insufficient sleep	Difference between reported need and the realisation of sleep ⩾2 h

*Expressed as absolute alcohol.

### Participants

The study was performed within one corporation in Finland. Inclusion criteria were permanent employment and age 18–60 years. Questionnaires were sent to a cohort of 3115 employees in September 2004. The proposed study design, implications of the trial, and alternative options were explained in the cover letter. The letter also emphasised that taking part in the trial was voluntary, and employees would get the best treatment available and full attention of the occupational physician even if they did not want to participate. In addition, it was explained that participants were free to withdraw from the trial at any point, and it would not prejudice their treatment. Of the target group, 49% were employed in the construction industry: civil engineering, building contracting, technical building services and building materials industry. 51% were employed in the repair, service and maintenance of buildings, industrial installations or communications networks.

### Randomisation

After collecting all responses and processing the risk group classification, a research assistant randomised each subject in the HR and IR groups into one of the two subgroups, intervention and control (“high risk”: HR-IG and HR-CG; “intermediate risk”: IR-IG and IR-CG). First, to ensure a balanced distribution of subjects by age, scripted four-digit identification codes (ID) were sorted by age within both RCTs and then all other items but the ID codes were removed from the list of subjects. An IT expert did this first step. After that the research assistant performed the randomisation in blocks of 10. A biostatistician had prepared the order from a random number table. The research assistant and researchers were not aware of which of the codes belonged to the intervention group and which to the control group in either trial. Neither were they able to identify the individuals based on the IDs, and could not therefore predict the group assignments. The coding was opened only after the primary analysis of the follow-up data was completed.

### Intervention versus care as usual

#### RCT 1: High risk group

The employees’ own occupational nurses and physicians carried out the intervention for subjects at HR. Forty eight occupational health centres were involved in the study.

The employees in the HR-IG received a letter with personal feedback of their questionnaire results and invitation to a consultation at the occupational health services (OHS). At most, two reminders were sent. The main purpose of the consultation was the construction of an action plan, and if appropriate, referral to a further consultation by a specialist, or psychologist. The occupational nurse first started the consultation, the planned duration of which was 90 minutes, and an occupational physician joined the meeting later if needed. The individual findings of the questionnaire were available for the OHS professionals during the consultation. Key treatment processes were defined in advance and the policies and practices at the occupational health centres were not altered as a result of the study.

To find out what actions were taken within the intervention, an occupational nurse wrote a personal file for each employee in the HR-IG at the end of the follow-up. The personal files included information about the employee attending to the consultation, the referrals to further evaluation or interventions, the health advice received at the OHS, the considerations of OHS professionals that no further actions were needed, and the refusals of some employees to take further action. Additionally, the nurses reported if the employee had already received treatment at the OHS for the health issues that were the reason for the invitation of consultation.

The employees in the HR-CG received care as usual. They could consult their occupational nurse or physician on request, but they were not invited for a consultation and did not receive feedback of their results.

#### RCT 2: Intermediate risk group

The intervention for workers at IR consisted of an access to medical counselling over the telephone from one phone advice centre. The employees in the IR-IG received a letter with personal feedback of their results and invitation to call the phone advice centre in order to receive respective medical advice. Two reminders were sent. The switchboard was always open, and the cost for the telephone call was the same as for a local call. All telephones were manned by trained nurses with several years of experience and specific training for their job. During the counselling the individual findings of the questionnaire were available for the nurses who also had access to relevant health databases while providing the health advice. The employees in the IR-CG received care as usual.

### Measurements

#### Questionnaire data

The baseline questionnaire included items on the following: lifestyle, anthropometrics, sleep disturbances, work-related stress and fatigue, depression, pain, disability due to musculoskeletal problems, and a prediction of future working ability. The responses were interpreted on the basis of a priori defined cut-off limits (table 1).

#### Sickness absence from work

Sickness absence data were obtained, without medical diagnoses, from the employer’s records. The baseline covered the period from 1 October 2003 to 30 September 2004 and the follow-up covered the period from 1 October 2004 to 30 September 2005. Data privacy was strictly followed. Records were checked for inconsistencies. Maternity/paternity leave and absence from work to care for a sick child are not included in the sickness absences.

### Sample size calculations

#### RCT 1: High risk group

The target sample size of 420 employees was based on the assumptions that 360 of them can be followed-up for one year, and that there will be a 15% difference between the groups in sickness absence with the mean baseline sickness absence estimated to be 20 (SD 9) days/year. Assuming a normal distribution for the outcome variable this gave an alpha of 0.05 with 80% power.

#### RCT 2: Intermediate risk group

The original target sample size of 840 employees was based on the assumptions that 686 of them can be followed-up for one year, and that there will be an 11% difference between the groups in sickness absence with the mean baseline sickness absence estimated to be 11 (SD 5) days/year. This gave an alpha of 0.05 with 80% power. However, at the time of randomisation, there were only 537 subjects eligible for the IR group. We reviewed the power calculation: our sample size was sufficient to detect a 14% difference.

### Statistical methods

We carried out an intention-to-treat analysis. The effectiveness of the interventions was estimated by the difference of mean number of sickness absence days between the randomised groups, and the confidence interval was computed based on t distribution.^26^ We used Statistica data analysis software, version 6 (StatSoft, Inc, Tulsa, OK, USA; 2001).

## RESULTS

**Figure 1:**
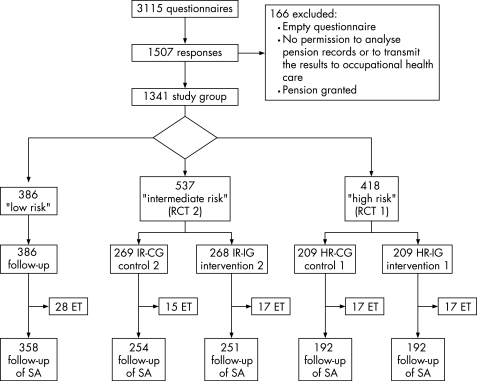
Study flow. ET, employment terminated during follow-up, SA, sickness absence.

At baseline, we received 1507 responses (48.4%) of which 166 were excluded. Thus, the final study population consisted of 1341 subjects (fig 1). The respondents were on average 44 years old (range 19–61 years). Of them 12% were females and 62% were blue-collar workers.

### Risk classification

418 (31%) subjects belonged to the high risk group, 537 (40%) to the intermediate risk group, and 386 (29%) to the low risk group. In comparison to other participants, the subjects in the high risk group were on average older and a larger proportion of them was male and worked in physically demanding jobs (table 2).

**Table 2:** Baseline characteristics, and one-year follow-up of sickness absence in risk groups of work disability

	Risk group classification			All subjects
	Low risk	Intermediate risk	High risk	
*Baseline*				
n	386	537	418	1341
Mean age (years) (range)	43 (19–60)	43 (19–61)	47 (23–60)	44 (19–61)
Sex (female; %)	19	12	6	12
Blue-collar (%)	52	58	78	62
Sickness absence				
None (% within group)	46	49	30	42
Mean (days)	5.6	5.3	18.8	9.6
Standard deviation	13.9	10.6	36.6	23.6
Median (days)	1	1	5	2
Upper quartile (days)	6	5	19	9
Maximum (days)	145	72	229	229
Sum (days)	2156	2827	7854	12837
Employment terminated during follow-up (%)	7	6	8	7
*1 year follow-up*				
n	358	505	384	1247
Sickness absence				
None (% within group)	46	45	27	40
Mean (days)	6.1	6.9	24.6	12.1
Standard deviation	13.1	13.4	49.1	30.5
Median (days)	1	1	6	2
Upper quartile (days)	6	7	21	10
Maximum (days)	107	115	365	365
Sum (days)	2181	3506	9446	15132

The risk classification predicted sickness absence: 62% of sickness absence days during the 12-month follow-up took place within the high risk group (table 2).

### Effectiveness of the interventions

#### RCT 1

The occupational health intervention for the HR-IG was effective in controlling sickness absence. In the HR-CG, the mean, median and total sickness absence days increased, and the proportion of subjects with zero absence decreased. No change took place in the mean, median and total sickness absence days, or in the proportion of subjects with zero absence in the HR-IG (table 3). The group difference between the means was 11 days (95% CI 1 to 20).

**Table 3:** Baseline characteristics and sickness absence in the intervention and control (usual care) groups in RCT 1 (occupational health intervention for the high risk group) and RCT 2 (telephone advice for the intermediate risk group)

	Group allocation			
	Intermediate risk (IR)		High risk (HR)	
	Control (IR-CG)	Intervention (IR-IG)	Control (HR-CG)	Intervention (HR-IG)
*Baseline*				
n	269	268	209	209
Mean age (years)	42.9	42.8	46.8	46.7
Sex (female; %)	12	13	6	6
Blue-collar (%)	57	58	80	77
Sickness absence				
None (% within group)	60	55	43	34
Mean (days)	4.6	5.9	17.9	19.7
Standard deviation	9.5	11.5	36.3	37.0
Median (days)	0	1	4	6
Upper quartile (days)	5	6	18	20
Maximum (days)	72	70	229	221
Sum (days)	1246	1581	3736	4115
Employment terminated during follow-up (%)	5.6	6.3	8.1	8.1
*1 year follow-up*				
n	254	251	192	192
Sickness absence				
None (% within group)	46	45	23	31
Mean (days)	6.9	7.0	29.9	19.3
Standard deviation	14.3	12.4	53.3	44.0
Median (days)	1	2	9	5
Upper quartile (days)	7	7	32	15
Maximum (days)	115	73	286	365
Sum (days)	1755	1751	5744	3702

#### RCT 2

The occupational health intervention by phone advice for the IR-IG was not effective in reducing sickness absence. The mean, median and total sickness absence days, or the proportion of subjects with zero absence did not differ between the IR-CG and IR-IG (table 3).

### Adherence

**Figure 2:**
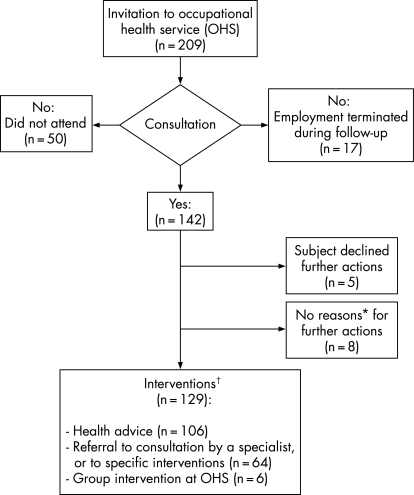
Adherence to the occupational health intervention: HR-IG (RCT 1). *According to the OHS professionals. †Many subjects received more than one intervention.

Of the subjects in the HR-IG in the RCT 1 (n = 209), 142 (68%) attended the consultation at OHS (fig 2). Fifty did not attend for unknown reasons. The employment had terminated with 17 subjects during the follow-up. Of the attendees, five refused further examinations or interventions, and the OHS professionals had considered that eight subjects did not warrant further actions. 129 subjects ended up in the interventions: health advice (n = 106), referral to consultation or hospital outpatient clinic (n = 64), or a group intervention at the OHS (n = 6), in different combinations. Of the 142 subjects who visited OHS, 72 (51%) had not received earlier treatment at OHS for the respective reasons for belonging to the high risk group.

Of the subjects in the IR-IG in the RCT 2 (n = 268), 57 (21%) had called the phone advice centre during the follow-up.

### Adverse events

No adverse events were reported during the interventions.

## DISCUSSION

### Main findings

It was possible to identify a group of employees with high risk of work disability and subsequent sickness absence. Moreover, the occupational health intervention was effective in controlling sickness absence within this group. The difference compared with the usual care treatment arm was 11 days per year, which is obviously of economic importance. The majority of subjects in the intermediate risk intervention did not use the telephone health advice and its effectiveness remains uncertain.

### Strengths and weaknesses of the study

The main strength of this study lies in the pragmatic approach in the randomised controlled trial for employees at high risk. All permanent employees in the target cohort were offered the opportunity to participate. Although the response rate was somewhat low, it was in line with other studies in occupational populations in European countries.^27^ The employees’ own occupational nurses and physicians carried out the occupational health intervention. Adherence within the HR-IG was reasonably high, and the intervention succeeded in capturing many workers with underlying health problems that had not been properly attended to. Of the subjects who visited OHS, more than half had not received treatment at OHS before. New ways of treatment were not introduced and the intervention relied on existing practices.

As there was no initial randomisation to getting a screening questionnaire or not, our study cannot genuinely answer the overall question of whether the screening programme as a whole was effective. Our research question was formulated to study whether the interventions for the “high risk” and “intermediate risk” groups were effective. However, it is possible to estimate whether the savings due to the reduction of sickness absence in the “high risk” group exceed the investment in the whole screening process, but we leave the calculation to be done by the reader with the reader’s own cost parameters.

Control arm contamination—that is, subjects randomised to usual care receiving the same treatment as the intervention subjects—is a common source of bias in randomised trials. In the present trial randomisation within the high risk and intermediate risk groups was performed individually, instead of clusters. Theoretically, control arm contamination within workplaces is a potential weakness in both trials in such setting. However, the subjects randomised to the usual care at OHS did not receive any feedback of their own survey results—that is, in which group they belonged. Moreover, most of the subjects were working in small workplaces or workgroups and therefore interaction between treatment arms would have been limited anyway: there were 48 occupational health centres involved in the study and far more workplaces. Furthermore, the OHS personnel were not aware of the survey results of the usual care groups and thus could not offer them the same services as for the intervention group. For these reasons, we believe that it is unlikely that the results would be biased by contamination.

Our primary outcome was based on recorded sickness absences. This has several advantages: good coverage, accuracy and consistency.^28^ We were able to follow 92% of the subjects in both arms of the high risk trial. Despite the heavily skewed distribution of sickness absence, in sufficiently large samples linear regression models, including t test and ANCOVA, are valid for any distribution.^26^

### Some differences compared with previous studies

The majority of previous randomised controlled trials in occupational health settings have been illness-related, or the focus has been on highly selected groups of employees. Some RCTs for musculoskeletal disorders^8^^–^10 and depression^11^ have been reported. Few studies have dealt with developing a screening instrument for employees at high risk of work disability and sickness absence.^7^ ^15^ Two RCTs have aimed at reducing sickness absence within a high-risk subgroup.^16^ ^17^ Fleten and Johnsen reported a trial in Norway in 1997–8 with 990 consecutive newly sick-listed employees with musculoskeletal or mental disorders randomised to intervention and control group.^16^ The minimal postal intervention—a general information letter—reduced the length of sick leave periods in subgroups with mental disorders, rheumatic disorders and arthritis, but did not show efficacy in the intention-to-treat analysis. A Dutch trial^17^ within one large company randomised 116 employees to an intervention (n = 61) and control (n = 55) group. Subjects were older than 50 years and had reported that they would not be able to continue in the present job until retirement. The programme was executed by an occupational physician and comprised at least three consultations including an assessment interview. The procedure included the construction of a detailed action plan, consultation of the employee’s supervisors and personnel managers and, if appropriate, referral to the general practitioner, a medical specialist or psychologist. The authors reported fewer retirements in the intervention group (11%) than in the control group (28%). The total average number of sick leave days in two years was 82 for the intervention group and 108 for the control group.

## CONCLUSIONS

Employees at a high risk for sickness absence can be identified by a health survey and occupational health care can support the working ability of these individuals. The intervention showed a clear advantage in sickness absences in comparison with usual care, but the cost consequences of intervention and usual care need to be considered in order to evaluate the cost effectiveness of occupational health intervention in the high risk group. Future research should also address the question of whether the same intervention approach is effective in different occupational settings and professional groups.

**Main messages**

- It was possible to identify individuals at a high risk of sickness absence with a simple health questionnaire among employees predominantly engaged in physical work.
- An occupational health intervention, which included an invitation to occupational health service for a consultation and, if appropriate, a referral to specialist treatment, was effective in controlling sickness absence within the high risk group.
- The mean difference of 11 days between the intervention and usual care treatment arms in this randomised trial is likely to be economically advantageous.

**Policy implications**

- Identification of individuals at high risk of sickness absence can be done with self-administered health questionnaires.
- Occupational health intervention can control sickness absence within the high risk group.
